# Integrative modeling of transcriptional regulation in response to antirheumatic therapy

**DOI:** 10.1186/1471-2105-10-262

**Published:** 2009-08-24

**Authors:** Michael Hecker, Robert Hermann Goertsches, Robby Engelmann, Hans-Juergen Thiesen, Reinhard Guthke

**Affiliations:** 1 Leibniz Institute for Natural Product Research and Infection Biology – Hans-Knoell-Institute, Beutenbergstr. 11a, D-07745 Jena, Germany; 2 University of Rostock, Institute of Immunology, Schillingallee 70, D-18055 Rostock, Germany

## Abstract

**Background:**

The investigation of gene regulatory networks is an important issue in molecular systems biology and significant progress has been made by combining different types of biological data. The purpose of this study was to characterize the transcriptional program induced by etanercept therapy in patients with rheumatoid arthritis (RA). Etanercept is known to reduce disease symptoms and progression in RA, but the underlying molecular mechanisms have not been fully elucidated.

**Results:**

Using a DNA microarray dataset providing genome-wide expression profiles of 19 RA patients within the first week of therapy we identified significant transcriptional changes in 83 genes. Most of these genes are known to control the human body's immune response. A novel algorithm called TILAR was then applied to construct a linear network model of the genes' regulatory interactions. The inference method derives a model from the data based on the Least Angle Regression while incorporating DNA-binding site information. As a result we obtained a scale-free network that exhibits a self-regulating and highly parallel architecture, and reflects the pleiotropic immunological role of the therapeutic target TNF-alpha. Moreover, we could show that our integrative modeling strategy performs much better than algorithms using gene expression data alone.

**Conclusion:**

We present TILAR, a method to deduce gene regulatory interactions from gene expression data by integrating information on transcription factor binding sites. The inferred network uncovers gene regulatory effects in response to etanercept and thus provides useful hypotheses about the drug's mechanisms of action.

## Background

The molecular interactions within a biological system give rise to the function and behavior of that system. In systems biology, one aims to formulate the complex interactions of biological processes by mathematical models. A major focus of the field is the uncovering of the dynamic and intertwined nature of gene regulation.

Gene expression is mainly regulated at the level of mRNA transcription by proteins called transcription factors (TFs), that specifically bind the DNA at the regulatory region of their target genes. A number of collections of experimentally defined TF binding sites (TFBS) have been assembled. The most commonly used is the Transfac database, which catalogs eukaryotic TFs and their known binding sites [1]. The expression level of a gene usually depends on the occupancy states of multiple TFBS. However, gene regulation is much more complex and includes different layers of post-transcriptional control. The entirety of gene regulatory processes constitutes a network of genes, regulators, and the regulatory connections between them – namely a gene regulatory network (GRN). In the past, various modeling approaches have been proposed to (partially) reconstruct GRNs from experimental data on the basis of different mathematical concepts and learning strategies, and distinct degrees of abstraction [2-4]. A graph is always the basic modeling scheme for a GRN, with nodes symbolizing regulatory elements (e.g. genes and proteins) and edges representing (activatory and inhibitory) relationships between them. Common mathematical formalisms of such a graph are Boolean networks, Bayesian networks, association networks and systems of equations. Boolean networks assume that genes are simply on or off, and apply Boolean logic to model dynamic regulatory effects. In contrast, Bayesian networks model gene expression by random variables and quantify interactions by conditional probabilities. Interactions in association networks are typically undirected and derived by analyzing pairs of genes for co-expression e.g. using mutual information as a similarity measure. Systems of equations describe each gene's expression level as a function of the levels of its putative predictors. For specific types of functions they could draw on well developed statistical techniques to efficiently fit their model parameters. However, GRN inference is always a challenging task because of incomplete knowledge of the molecules involved, the combinatorial nature of the problem and the fact, that often available data are limited and inaccurate. Microarray gene expression data are typically used to derive rather phenomenological GRN models of how the expression level of a gene is influenced by the expression level of other genes, i.e. the model also includes indirect regulatory mechanisms. Obviously, the incorporation of other types of data in addition to gene expression data (e.g. gene functional annotations, genome sequence data, protein-protein and protein-DNA interaction data) as well as the integration of prior biological knowledge (e.g. from scientific literature) supports the inference process. Moreover, it is necessary to utilize biological plausible assumptions considering the network topology (e.g. structural sparseness). The integration of diverse types of biological information and modeling constraints allows for more accurate GRN models and is a current challenge in network reconstruction. Bayesian networks and systems of linear equations have been most studied for such combined analyses [3-5].

Organizing biological data in network models may help understanding complex diseases such as human autoimmune diseases [6]. Many studies implicate hundreds of genes in the pathogenesis of autoimmune diseases, but we still lack a comprehensive conception of how autoimmunity arises. Understanding structure and dynamics of molecular networks is critical to unravel such complex diseases. Network analyses may not only support the investigation of autoimmune diseases but also the optimization of their treatment. Here, we focus on rheumatoid arthritis (RA), which is a multifactorial polygenic disease and might be termed a systems biology disease. RA is a chronic inflammatory disorder primarily afflicting the synovial joints, and autoimmunity plays a pivotal role in its chronicity and progression. The disease is characterized by autoreactive behavior of immune cells and the induction of enzymes which lead to the destruction of cartilage and bone [7]. The inflammatory processes are triggered by cytokines and other immune system-related genes that form a complex network of intra- and intercellular molecular interactions. A number of cytokine proteins play a critical role as mediators of immune regulation. In RA, the two cytokines TNF-alpha and IL-1 are considered master regulators that act in a complementary and synergistic manner [8,9]. By blocking TNF-alpha, etanercept intervenes this molecular network and thus is thought to re-balance the immune system's dysregulation [10-12]. Etanercept therapy in RA patients has been proven to slow disease progression, but the precise molecular mechanisms remained unclear. To investigate the therapeutic effects on transcriptional regulation, GRN inference techniques can be applied. This could lead to a better understanding of the modes of action of etanercept as well as the pathogenesis underlying RA. We may also understand why the drug fails to control the disease in about 30% of the patients (non-responders).

**Figure 1:**
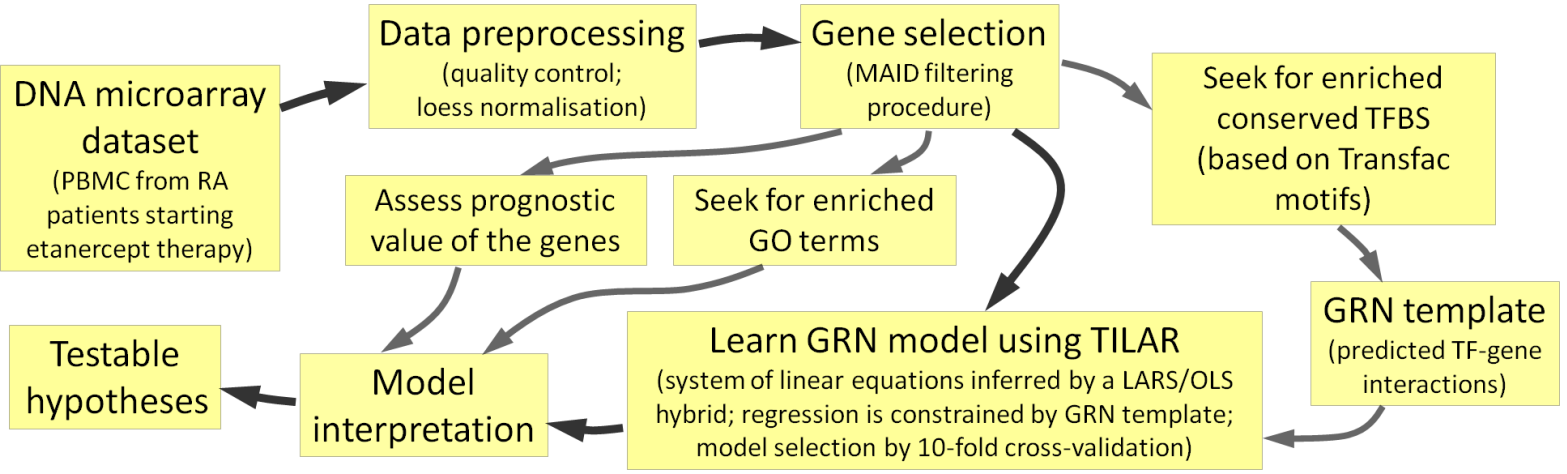
**Workflow used to study gene regulatory effects in response to etanercept therapy**. A network model of transcriptional regulation is inferred by integrating transcription factor binding site information.

We studied a group of 19 patients suffering from RA for which DNA microarrays were used to obtain genome-wide transcriptional profiles within the first week of etanercept administration [13]. A set of etanercept responsive genes was attained. The majority of these genes are known to control the body's immune response. Several TFBS were identified as overrepresented in the genes' regulatory regions and we used the corresponding information on TF-gene interactions as a template for modeling the underlying GRN. A system of linear equations was chosen to mathematically describe the regulatory effects between the genes and TFs (i.e. the network nodes). We used a hybrid of the Least Angle Regression (LARS) and the Ordinary Least Squares regression (OLS) to find the model structure and estimate the coefficients. In doing so, the modeling is constrained to include only a subset of the putative TF-gene interactions. That way, our approach considers that genes usually regulate other genes indirectly through the activity of one or more TFs, which makes the model straightforward to interpret in terms of true molecular interactions. The resulting GRN was further analyzed using e.g. gene ontology (GO) and clinical information (figure 1). We found that our integrative modeling strategy, namely the TFBS-integrating LARS (TILAR), is able to reconstruct GRNs more reliably than other established methods. This is one of the first studies that utilizes network analysis to investigate transcriptional regulation in response to a therapeutic drug in humans [14].

## Results and discussion

### Effects of etanercept therapy on gene expression

We used the Affymetrix microarray dataset from Koczan *et al*. [13] which provided expression levels of peripheral blood mononuclear cells (PBMC) measured in 19 patients suffering from RA. For each patient, blood samples were taken before treatment (baseline) as well as 72 (day 3) and 144 hours (day 6) after start of immunotherapy by etanercept. Clinical response was assessed over 3 months and revealed 7 patients with persistent disease activity (non-responders).

We analyzed the DNA microarray data in respect to common gene expression changes observed in the whole group of patients after therapy onset. First of all, we pre-processed the data to correct for systematic effects. More importantly, signal intensities were calculated by applying a custom chip definition file by Ferrari *et al*. that is composed of custom-probesets including only probes matching a single gene [15]. As such, a one-to-one correspondence between genes and custom-probesets is preserved, which deeply improves gene-centered analysis of human Affymetrix data [16]. Finally, the data pre-processing yields expression levels of 11,174 different genes for each of the 55 microarrays in the dataset (for details see the methods section).

Afterwards, we identified a set of genes significantly up- or down-regulated in response to etanercept. It is important to note that the filtering of genes is a crucial step in GRN inference as there is a tight relationship between model complexity (i.e. network size and level of detail of the model), the amount of data required for inference and the quality of the results. On the one hand, a small and detailed network model might better fit the given data, but only a sufficiently large model can capture the fundamental properties that constitute a GRN including scale-freeness, redundancy and self-regulation. In this study, we utilized a *t*-statistic in conjunction with an MA-plot-based signal intensity-dependent fold-change criterion (referred to as MAID filtering) to select genes with expression changes in the first week of therapy (see methods). Through this filtering we identified 37 genes as differentially expressed at day 3 versus baseline, and 57 genes at day 6. Altogether, 48 genes were found down-regulated and 35 genes up-regulated, comprising a set of 83 genes in total (additional files 1 and 2).

We searched for overrepresented terms of the GO biological process ontology in the list of 83 selected genes and found that most of the genes are known to control the body's immune response (additional file 3, see methods). Remarkably, genes of the I-kappaB kinase/NF-kappaB cascade (GO:0007249) are enriched in the gene set and represented by 5 genes (NFKBIA, TNFRSF1A, TLR8, NOD2, HMOX1). NF-kappaB is a key factor in the transcription of many inflammatory genes and has been implicated in the pathological processes of RA. The NF-kappaB cascade is mainly activated by the proinflammatory cytokines IL-1 and TNF-alpha. As was shown, TNF-blocking agents such as etanercept prevent TNF-alpha from binding to its receptors, induction of signal transduction cascades and activation of TFs including NF-kappaB [10]. Here, we found NFKBIA, that inactivates NF-kappaB by trapping it in the cytoplasm, up-regulated early after therapy initiation. On the other hand, genes known to activate the NF-kappaB protein, e.g. NOD2 and TNFRSF1A (a TNF receptor), were down-regulated after therapy onset. Thus, the result of our filtering indicates the expected suppression of NF-kappaB activity by etanercept. In addition, we found evidence for a modulation of B cell mediated immunity. The corresponding GO category (GO:0019724) comprises the genes C1QB, CLU and TLR8 whose mean expression was significantly lower at day 3 and 6 compared to baseline, respectively. Interestingly, TLR8 signaling is linked to the control of CD4+ regulatory T (Treg) cells. Treg cells actively suppress host immune responses and, as a consequence, play an important role in preventing autoimmunity [17]. TLR8 is thought to initiate immune processes by reversing the suppressive function of Treg cells [18]. Its down-regulation by etanercept might be an important factor to control the disease.

Genes responsive to etanercept administration are probably under control of certain TFs, whose activities are (maybe indirectly) affected by this drug. Therefore, we analyzed the regulatory regions around the respective transcription start sites (TSS) of these genes for occurrence of overrepresented TFBS (see methods). Identifying TFBS, particularly in higher eukaryotic genomes, is an enormous challenge and cross-species sequence conservation is often used as an effective filter to improve the predictions. We found evolutionarily conserved binding sites enriched for 12 TFs (represented by 19 Transfac binding profiles). These 12 TFs connect 52 out of the 83 genes through 96 TF-gene interactions, whereas each TF is linked to at least 4 genes (table 1). The list of TFs includes C/EBP-beta, which is an important transcriptional activator in the regulation of genes involved in immune and inflammatory responses, including the cytokines IL-6, IL-8 and TNF-alpha [19]. Binding sites for the TATA binding protein (TBP) were detected in 14 genes. TBP binds DNA at the TATA-element, and as a subunit of the TFIID complex coordinates the initiation of transcription by RNA polymerase. Although TBP is always involved, its TATA-binding activity is dispensable for the positioning of the RNA polymerase. In fact, approximately 76% of human core promoters lack TATA-like elements [20]. However, in the set of 83 genes, those genes having the TATA box were overrepresented. The two TFs ZIC1 and ZIC3 were considered as one TF entity, as they have highly similar DNA binding properties. None of the 12 TFs showed significant transcriptional changes in the data. Nevertheless, the information on predicted TF-gene interactions can be used as a GRN template during inference. Before describing how this is done by TILAR we will outline the general principles of the modeling approach.

**Table 1:** Evolutionarily conserved binding sites were found to be enriched for 12 TFs.

**TF Name**	**Transfac ID**	**Official Full Name**	**P-value**	**Expected Count**	**Count**
TBP, TFIID	V$TBP_01, V$TATA_C, V$TATA_01	TATA box binding protein	0.0042	6.41	14
C/EBPbeta	V$CEBPB_01, V$CEBPB_02	CCAAT/enhancer binding protein beta	0.0112	5.03	11
Zic1, Zic3	V$ZIC1_01, V$ZIC3_01	Zic family member 1/3	0.0183	6.13	12
AP-2rep	V$AP2REP_01	Kruppel-like factor 12	0.0264	1.68	5
HNF-1, HNF-1A	V$HNF1_01, V$HNF1_C	HNF1 homeobox A	0.0274	2.30	6
Lmo2	V$LMO2COM_01, V$LMO2COM_02	LIM domain only 2	0.0352	5.98	11
SRY	V$SRY_02	sex determining region Y	0.0374	1.85	5
ATF-2	V$CREBP1_01	activating transcription factor 2	0.0408	1.30	4
Cart-1	V$CART1_01	ALX homeobox 1	0.0415	1.30	4
COMP1	V$COMP1_01	cooperates with myogenic proteins 1	0.0422	3.23	7
Hlf	V$HLF_01	hepatic leukemia factor	0.0470	1.97	5
NF-1, NF-1/L	V$MYOGNF1_01, V$NF1_Q6	nuclear factor I	0.0492	7.10	12

					**Σ = 96**

The column "Count" denotes the number of genes that possess a TFBS for the respective TF. All in all, 96 TF-gene interactions were predicted (GRN template).

### Linear network modeling

We chose a system of equations to model the regulatory interactions among the genes affected by etanercept therapy. The concept of modeling gene regulation by a system of equations is to approximate gene expression levels as a function of the expression of other genes and environmental factors. Modeling GRNs by systems of equations has several benefits as they can describe regulatory effects in a flexible, quantitative, directed manner, and take into account that gene regulators act in combination. With systems of equations one can easily model positive and negative feedback loops, and describe even non-linear and dynamic phenomena of biological systems. However, as more complex models require higher amounts of accurate data to learn their parameters reliably, researchers often utilize systems of linear equations (linear models). Linear models have been successfully employed in many applications, e.g. to reconstruct GRNs relevant for development of the central nervous system in rats [21], osteoblast differentiation in mice [22,23], galactose regulation in yeast [24] and immune response of human blood cells to bacterial infection [25]. Linear models assume that gene regulatory effects are limited to be linear and additive and a simple one can be written as:

(1)

where vector *x*_*i *_contains the *M *expression levels measured for gene *i*, *N *is the number of genes in the network, and the weights *w*_*ij *_define relationships between the genes. When inferring a linear model we need to estimate the weights *w*_*ij *_(i.e. the model parameters) from the data. The weights specify the existence of regulatory relationships between genes, their nature (activation or inhibition) and relative strength. If *w*_*ij *_> 0 gene *x*_*j *_activates gene *x*_*i*_, if *w*_*ij *_< 0 *x*_*j *_inhibits *x*_*i*_, and *w*_*ij *_= 0 implies that *x*_*i *_is not under control of *x*_*j*_. This simplicity makes linear models easy to interpret, even if the encoded relationships have a wide range of meanings: edges in the network might represent direct physical interactions (e.g. when a gene encodes a TF regulating another gene) or rather conceptual interactions (e.g. when the expression levels of two genes merely correlate).

Linear models can also be used to describe the dynamics of the network. In this case, the model is a system of linear difference equations that approximates the change of gene expression in time. However, this approach is inappropriate for our application as the time-series in the microarray dataset consist of only 3 time-points and the time between two subsequent measurements is rather long (3 days). Nevertheless, the modeling strategy illustrated here can be easily adapted for the inference of dynamic models.

To fit the (static) linear model to the data, equation (1) can be written in matrix form as follows:

(2)

These *N *systems can be coupled as:

(3)

Now, a GRN model can be inferred by estimating *β *(comprising all the model parameters in *w*) from input matrix ***X ***(having *M' *= *MN *rows and *N' *= (*N*-1)*N *columns) and output vector ***y ***using OLS regression.

However, despite the fact that linear models are a strong simplification of the true GRN, equation (3) is already an underdetermined system of linear equations in our particular study as the number of genes in the network (*N *= 83) is greater than the number of measurements (*M *= 55). That means, infinitely many solutions exist. Therefore, biologically motivated constraints have to be included to tackle this problem. The most commonly used modeling constraint is the sparseness of GRNs. Sparseness reflects the fact that genes are regulated only by a limited number of regulators. The sparseness constraint minimizes the number of edges, i.e. reduces the effective number of model parameters. Sparse linear models can be reconstructed via the Lasso (Least absolute shrinkage and selection operator) method [26], which effectively performs simultaneous parameter estimation and variable selection. The Lasso is a version of OLS that constrains the sum of the absolute regression coefficients *β*:

(4)

The Lasso penalizes model complexity by shrinking the coefficients *β*_*j *_(and hence *w*_*ij*_) toward 0, more so for small values of *s*. A modification of the LARS algorithm implements the Lasso [27]. LARS builds up estimates for *β *in successive steps, each step adding one covariate to the model, so that gradually model parameters are set non-zero. In simple terms, LARS is a less greedy version of traditional forward selection methods. LARS and its variants are computationally efficient. The algorithm requires only the same order of magnitude of computational effort as OLS to calculate the full set of Lasso estimates (i.e. for all *s *≥ 0).

The Lasso approach was first introduced to infer regulatory interactions by van Someren *et al*. [28] and has since been applied in several GRN studies [22,29]. However, even if the network connectivity is constrained, there is a limitation in inferring GRNs using gene expression data only. Hence, there is a need to incorporate different types of information during network reconstruction. Various data and information from biomedical literature and databases can be utilized in combination with gene expression levels to increase model accuracy.

An integrative learning strategy usually consists of two steps. First, a template of the network is built, e.g. based on known TF-DNA interactions or molecular interactions automatically extracted from the literature by text mining. This template represents a supposition of the true network structure, that might be uncertain and incomplete. Second, an inference algorithm is applied that fits the model to the measured data while taking the template into account, trading off data-fit and template-fit. When inferring linear models, such template information can be included by adapting the Lasso method. This is possible by introducing additional weights *δ *on the coefficients *β *of the constraint in equation (4):

(5)

A relatively low weight *δ*_*j *_provokes that the edge corresponding to *β*_*j *_is preferred to be in the final model. Hence, the modeler is able to incorporate partial prior knowledge by setting the weights *δ*appropriately. Recently, this concept was applied to integrate human microarray data with regulatory relationships obtained by literature mining by defining each *δ*_*j *_as a constant [23] and as weight function of *β*_*j *_[13], respectively.

### TILAR – a TFBS-integrating linear modeling approach

**Figure 2:**
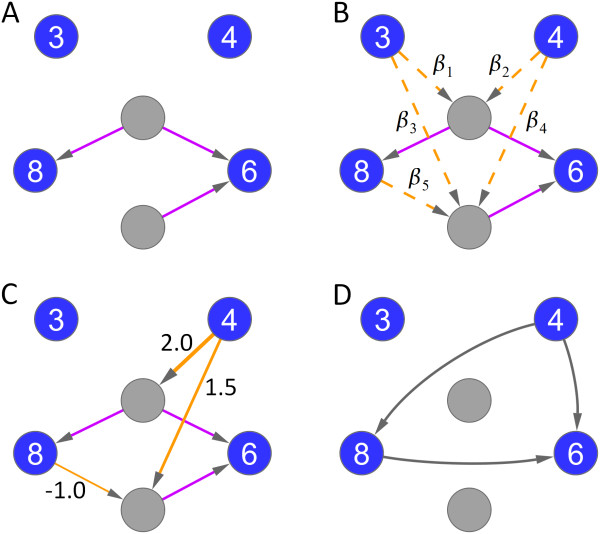
**Illustration of our proposed modeling conception**. Here, we aim to reconstruct a gene regulatory network consisting of 4 genes (dark blue) and 2 transcription factors (light gray). For simplicity, we assume that only one gene expression measurement was performed. The expression level of each gene is given in the gene nodes. (**A**) The GRN template: In this example, two genes possess at least one TF binding site in their regulatory region as indicated by 3 TF-gene interactions (purple). (**B**) In that case, there are 5 possible gene-TF interactions (i.e. model parameters *β*) in the network (dashed, orange). If available, we might consider prior knowledge on gene-TF interactions during inference (adaptive TILAR). (**C**) A possible inference result including 3 gene-TF interactions (solid, orange). Here, the model perfectly fits the data (e.g. "8" = 2.0·"4") with two nominal model parameters set to zero. (**D**) We can use the inferred model to derive gene-gene relationships from the edges between genes and TFs (gray). The benchmarking was conducted on such gene-gene interactions.

Here, we propose TILAR – a TFBS-integrating inference technique that differs from the adaptive Lasso approach, and employs TF binding information as prior knowledge. As we will show, it is even possible to combine the adaptive Lasso and TILAR. According to our modeling scheme, we distinguish two types of network nodes: genes (that were selected for inferring regulatory relationships between them) and TFs (for which respective binding sites are overrepresented in the gene set). Expression levels of the gene set (that possibly includes genes encoding TFs) are required for the modeling. The algorithm then aims to assign (directed) TF-gene and gene-TF interactions (network edges). A TF-gene interaction represents a physical interaction, i.e. a TF binds the region that encompasses the TSS of a certain gene and thus regulates its transcription. In contrast, gene-TF interactions can have different meanings: the gene itself might encode a transcriptional regulator of the TF, or the gene product controls the activity of the TF at the proteomic level, or the gene triggers signaling cascades that affect the TF, etc. Using both types of interactions, the model reflects that genes regulate other genes indirectly through a combination of TFs (figure 2). As a reminder, the TFBS overrepresentation analysis revealed 96 putative TF-gene interactions. Now, the idea is to use this information as a GRN template by constraining the modeling to include only a subset of these TF-gene interactions. As the inference method not necessarily uses all the given TF-gene interactions, we consider the fact that they are computationally predicted and therefore not all of them might refer to biologically functional binding sites. In practice, the algorithm starts with the entire set of TF-gene interactions and then iteratively removes avoidable interactions through a backward stepwise selection procedure (see methods). The information on (the current set of included) TF-gene interactions is written in matrix *B*, which is defined as:

(6)

In our particular study, 96 entries in *B *were set to 1 in the first iteration. TILAR then assigns the parameters in the model to gene-TF interactions, as follows:

(7)

where *F *is the number of TFs. For modeling the transcriptional regulation in response to etanercept we have *N *= 83 genes, that showed significantly changed expression levels after therapy onset, and *F *= 12 TFs, whose binding sites are overrepresented in the regulatory regions of the selected genes. In the model, each gene can exhibit a regulatory effect on each TF, except those TFs that hold a TF-gene interaction to this gene (this restriction is dispensable when inferring a dynamic model). If *w*_*kj *_= 0, there is no gene-TF interaction between gene *j *and TF *k*. Otherwise, gene *j *controls the activity of TF *k *and thus regulates all the genes that possess a TFBS for TF *k*. In this case, the expression levels of gene *j *explain the expression of the genes regulated by TF *k*. Again, to infer the GRN model, we need to estimate the model parameters *w*_*kj *_from the gene expression data while constraining the model to be sparse. Similar to equation (3), we can couple the subsystems, as follows:

(8)

where *N*_*r *_is the number of genes possessing at least one overrepresented TFBS, and *#B *is the number of TF-gene interactions considered at the current iteration. The coefficients *β *of this equation now correspond to gene-TF interactions in the model. Finally, a sparse solution to equation (8) can be found using the Lasso according to equation (4).

The TILAR modeling approach proposed here is advantageous for several reasons. First, TF expression levels are not required, since the activity of TFs is modeled implicitly (like a hidden node). This is beneficial, as mRNA levels of TFs are often low and do not necessarily correlate with TF activity. In fact, TF proteins often need to be activated by phosphorylation. Second, the nominal number of model parameters *w *in equation (7) is generally lower than in equation (1). Therefore, our method tackles the problem of having too many parameters in comparison to limited amounts of experimental data. In our particular application, equation (8) is an overdetermined system of linear equations (as *M*·*N*_*r *_= 55·52 = 2860 >*F*·*N*-#*B *= 12·83-#*B *= 996-#*B*, #*B *≤ 96), i.e. we are able to infer a complex network of 83 genes (and 12 TFs) without being in conflict with the data requirements. Third, by using TF binding predictions as prior knowledge we can reconstruct GRNs more reliably. Besides, the inferred models are relatively easy to interpret. Finally, the integration of TFBS information is accomplished by simply specifying the regression equation (i.e. input matrix ***X ***and output vector ***y***) adequately. Therefore, we can combine the TILAR approach with the adaptive LARS, i.e. solve equation (8) according to equation (5) if prior knowledge on gene-TF interactions is available (adaptive TILAR).

### Modeling the gene regulatory response to etanercept

To examine the early transcriptional effects of etanercept we applied the TILAR algorithm to construct a GRN model on the basis of gene expression data and knowledge on TF-gene interactions obtained by TFBS analysis. For this purpose, the GRN inference problem was formulated according to equation (8). The essential part of our modeling approach is the LARS algorithm that is used to obtain all possible Lasso solutions for this linear regression equation.

TILAR iteratively applies LARS in a backward stepwise selection procedure in order to refuse TF-gene interactions that do not fit the data well (see methods). Hence, the learning strategy takes into account that the prediction of TFBS might be error-prone. In this study, 12 out of 96 predicted TF-gene interactions were discarded. These 12 interactions may result from false positive TFBS predictions, or the magnitude of the TF-gene interactions was not enough for being confirmed based on the gene expression levels.

**Figure 3:**
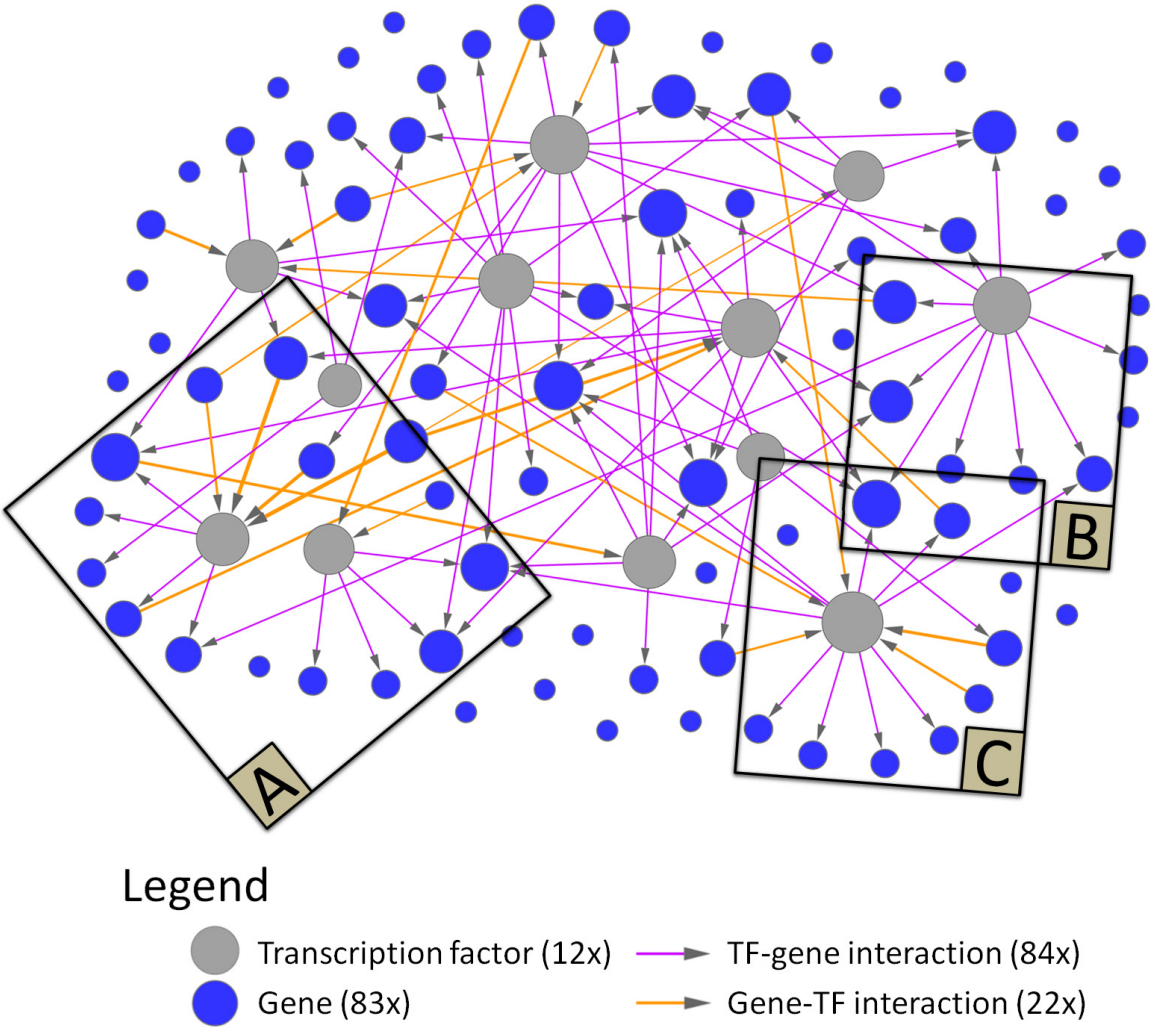
**Reconstructed gene regulatory network of genes up- or down-regulated during first week of therapy**. The TILAR algorithm used gene expression data and transcription factor binding predictions to infer a network of 84 TF-gene and 22 gene-TF interactions. The size of the nodes corresponds to their degree of connectivity. Three parts of the network model are shown in detail in figure 5. The full model is available as a Cytoscape session file of (additional file 5).

After we identified the subset of 84 TF-gene interactions, we used LARS to define which gene-TF interactions have to be included at different degrees of network connectivity. That means we used LARS only for variable selection, but the actual coefficients were estimated by OLS (see methods). This LARS/OLS hybrid technique usually achieves sparser estimates and more accurate predictions, and thus outperforms the ordinary Lasso [27,30]. Finally, we selected the most parsimonious estimate with low 10-fold cross-validation error (additional file 4). In this way, the method avoids overfitting to the data and consequently yields a sparse GRN model. The final model consists of 22 inferred gene-TF interactions and 84 TF-gene interactions, and was visualized using Cytoscape 2.6.0 (figure 3, additional file 5).

### Model interpretation

Systems biological models need to be interpretable in order to be useful. In general, the modeling goals of accurate prediction and interpretation are contradictory since interpretable models should be simple, but more accurate models might be quite complex. The model reconstructed here seems to satisfy both requirements. On the one hand, the network model is fairly complex as it consists of 95 nodes and 106 edges while using 22 model parameters *w*_*ij *_(specifying the strength of the gene-TF interactions). Yet the model is readily interpretable due to the intuitive linear modeling scheme.

**Figure 4:**
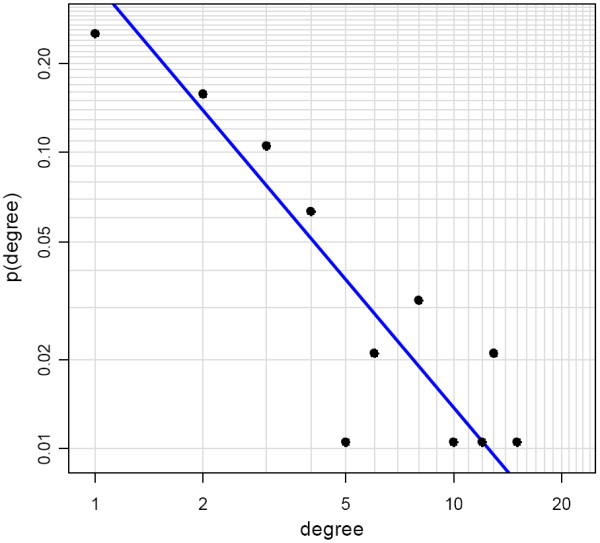
**Node degree distribution in log-log scale**. The network is scale-free, while transcription factors are more connected than genes. The orthogonal linear regression line is shown in blue.

Apparently, the inferred model is sparse, i.e. each network node is under control of only few regulators. The maximum in-degree in the GRN is 5 (on average 1.12). Nevertheless, some nodes (named hubs) are highly connected in the network, e.g. TBP which has an out-degree of 12. A further characteristic and biologically meaningful property of the network is its scale-free structure. Scale-freeness denotes the phenomenon that the degree distribution in biological networks often follows a power law, i.e. the fraction *P*(*k*) of nodes in the network having *k *connections goes as *P*(*k*)~*k*^-*γ*^, where *γ *is a constant. This means that in scale-free networks most of the nodes are lowly connected, while a few are relatively highly connected. Scale-freeness indicates a network's decentralization and structural stability, and in consequence its robustness against random fluctuations [31]. The scale-free design of GRNs is well studied in literature [32,33], and the GRN reconstructed here is scale-free with *γ *= 2.22 (as calculated according to Clauset *et al*. [34], see figure 4).

**Figure 5:**
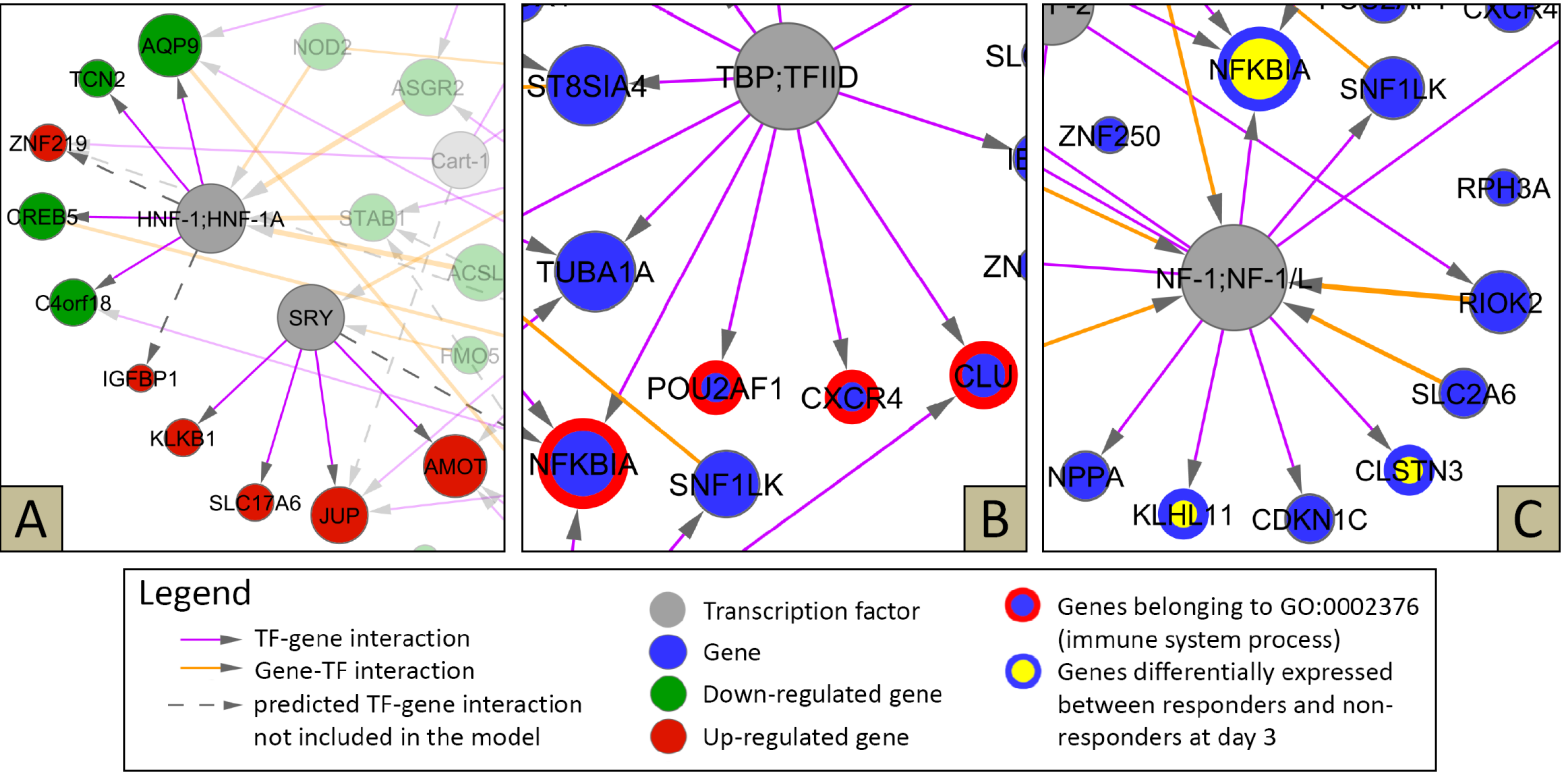
**Detail views of the network model shown in figure 3**. (**A**) The modeling strategy takes into account that target genes of a transcription factor are often co-expressed. For example, all the genes that are regulated by HNF-1 are down-regulated after therapy onset. Outer parts are shown with lower opacity. (**B**) A set of genes associated with the GO category "immune system process" is predicted to contain TATA-like elements in their regulatory regions. (**C**) Three genes were expressed lower in responders at day 3 and all of them are regulated by NF-1 according to the model.

A closer look at the interactions in the network revealed gene sets co-regulated by a common TF. For example, 6 TF-gene interactions were assigned to the transcriptional activator HNF-1 in the GRN template (table 1). Two of them were not considered in the final model as they were eliminated during backward stepwise selection. However, the 4 remaining genes that are predicted to be under control of HNF-1 (AQP9, TCN2, CREB5, C4orf18) are all down-regulated in the patients during first week of therapy (figure 5A). AQP9 is assumed to have some role in immunological response [35]. Hence, we can hypothesize that the activity of HNF-1 is lowered under etanercept therapy, which has (barely explored) effects on specific immune processes.

We also found that the network model highlights TFs that regulate functionally related genes (as annotated by GO). For instance, the model reveals TF-gene interactions of the transcription initiation factor TBP and the genes NFKBIA, POU2AF1, CXCR4 and CLU (figure 5B). These 4 genes not only share the TATA binding site in their regulatory region, but also belong to the same functional category (immune system process, GO:0002376). Nevertheless, they play different roles in inflammatory control. NFKBIA inhibits the activity of the NF-kappaB complex, which controls many genes involved in inflammation and is chronically active in RA [10]. Interestingly, the data show significantly elevated expression levels of NFKBIA in response to etanercept. POU2AF1 is a B cell-specific transcriptional co-activator that is known to stimulate immunoglobulin promoter activity [36], and CXCR4 is a chemokine (C-X-C motif) receptor that guides lymphocyte migration [37]. These findings suggest that the therapy by etanercept modulates the maladjusted immune system at multiple levels.

Other important features of a GRN are feedback and redundancy mechanisms. Regulatory feedback loops can be positive (i.e. reinforcing) or negative (i.e. self-balancing). Redundant links in the GRN allow genes to maintain their connection to other genes even if some genes are malfunctioning. Redundancy and self-control provide flexibility and adaptability to environmental changes, i.e. robustness against noise and failures [31]. An exemplary (positive) feedback loop in the inferred GRN model is the regulatory chain "CREB5 → C/EBP-beta → ASGR2 → HNF-1 → CREB5". Notably, C/EBP-beta encodes a TF that is important in the regulation of immune genes and has been shown to bind the regulatory regions of several cytokine and acute-phase genes. In RA, elevated levels of acute-phase proteins have been associated with progressive joint damage [38]. The feedback loop is finally formed by the two gene nodes CREB5 (which encodes a TF as well) and ASGR2. Both genes were down-regulated after therapy onset. Therefore, we assume that etanercept lowers the activity of C/EBP-beta while affecting a regulatory feedback mechanism.

The GRN model also contains a (positive) feedforward loop composed of the two ways "NOD2 → HNF-1" and "NOD2 → Lmo2 → STAB1 → HNF-1". NOD2 is a regulator of NF-kappaB activity [39] and was found down-regulated on days 3 and 6. The model predicts a gene-TF interaction between NOD2 and HNF-1, while we presume a decreased activity for HNF-1 as described previously. Alternatively, NOD2 is linked to LMO2, which has a crucial role in hematopoietic development and is connected to STAB1 according to the model. In turn, STAB1, a receptor which is supposed to function in angiogenesis and lymphocyte homing [40], has a gene-TF interaction to HNF-1, thereby closing the feedforward loop. Ultimately, this demonstrates the cooperative action of genes in the network.

As mentioned before, out of the 19 RA patients in the analyzed dataset 7 did not respond clinically to etanercept. Considering the potential side effects and the high costs of the therapy, the identification of patients who will most likely respond would contribute to a more optimized treatment of RA. To identify predictors (biomarkers) of the therapeutic outcome one might seek for differences in the gene expression of responder and non-responder patients before or early in therapy. The work by Koczan *et al*. is focused on this particular issue [13]. Here, we also compared the transcriptional levels of both patient groups using a two-sample *t*-test. At day 3, four network genes were found to be differentially expressed at the significance level *α *= 0.05 (additional file 1). Three of these genes (NFKBIA, KLHL11, CLSTN3) were expressed lower in the responder group and are regulated by a common TF node (NF-1) in the GRN model (figure 5C). NF-1 (nuclear factor I) constitutes a family of DNA-binding proteins with similar binding specificity, that participate in both cell type-specific transcription and replication [41]. Our model suggests that NF-1 regulates genes that are possibly relevant for the individual success of etanercept therapy, while the prognostic value of NFKBIA mRNA levels is already under discussion [13]. However, even if this hypothesis still has to be verified, the analysis clearly demonstrates the use of correlating clinical features with molecular network structures [6,42].

The GRN model provides many testable hypotheses and thus may be a starting point for new experiments. Those could aim to study the expression changes of specific genes in more detail, or to analyze their regulatory effects thereby validating parts of the inferred network. Take for instance the previously mentioned subnetwork of NF-1 and its target genes (figure 5C). The model suggests that members of the NF-1 family bind the promoters of 10 etanercept-responsive genes. This might be tested by electrophoretic mobility shift assays or chromatin immunoprecipitation techniques. One could also investigate the genes' transcriptional changes during therapy in a larger cohort of RA patients by generating expression profiles using real-time polymerase chain reaction. This would be particularly useful for the three genes that were found significantly lower expressed in the responder group. As a next step, levels of the respective proteins and protein isoforms could be quantified using western plots and enzyme-linked immunosorbent assays. For example, it would be interesting to measure the amount of HNF-1 proteins as we postulate a lowered activity of this TF as a molecular therapeutic effect. In the model all target genes of HNF-1 are down-regulated in response to etanercept administration (figure 5A). Similarly, other parts of the network are worth to study, e.g. the inferred regulatory feedback loop including C/EBP-beta and CREB5. Transcript and protein levels of *in vitro *cultures of PBMC cells may also be analyzed in a time-dependent manner. This allows for controlled perturbation experiments such as siRNA mediated knock-down of NF-1 expression with or without the presence of etanercept. Last but not least, one could examine the cell type-specific expression of genes in the network. Recent studies point out a functional impairment of Treg cells in RA [17]. It would be attractive to further elucidate the altered immunosuppressive capacity of Treg cells, their role in the treatment of RA and the modulation of Treg cells by Toll-like receptors such as TLR8 that was down-regulated in the dataset.

### Performance evaluation

To demonstrate the benefit of the TILAR modeling approach, we tried to evaluate how reliable the structure of the underlying GRN can be inferred. The assessment of the GRN inference performance is a challenging task, as evidently, true regulatory interactions are barely known and curated datasets for benchmarking are missing, though there are attempts to remedy this shortcoming [43,44]. A further difficulty is that the knowledge used to validate a GRN model must be different from the knowledge integrated during modeling.

Here, we utilized gene-gene interaction information obtained by text mining for performance evaluation (see methods). By assessing the inference quality on literature-derived (undirected) gene-gene links, we were also able to compare our method with other inference techniques which do not incorporate prior knowledge. It is important to note that regulatory gene-gene interactions are implicitly defined in our network model by gene-TF and TF-gene interactions, as genes are constrained to regulate other genes via one or more TFs (figure 2D). For the inferred network 158 gene-gene interactions can be deduced from the 22 gene-TF and 84 TF-gene edges. However, literature mining reports only 5 gene-gene relationships between the 83 genes in the network, which is not nearly enough for validation purposes. Since the biological role of many selected genes remains to be investigated, we assume that the lack of text mining information is mainly due to the literature bias, by which genes that have been intensively studied for many years (e.g. TNF-alpha) are cited more often than less prominent genes.

To overcome this issue, we sought for genes well described in the literature. For them we could expect many known gene regulatory interactions, so that a systematic evaluation of the performance of network reconstructions becomes feasible. We finally chose genes that are most frequently co-mentioned in the context of RA in PubMed. A respective list of genes was obtained from the Autoimmune Disease Database (version 1.2 as of August 19, 2008), which is a literature-based database that provides gene-disease associations of all known or suspected autoimmune diseases [45]. Out of the top 50 genes cataloged for the disease term "rheumatoid arthritis", 42 genes were measured in the Affymetrix dataset (additional file 6). We will denote the network of these 42 genes as the benchmarking GRN in the following.

Genes in the benchmarking network include several matrix-metallo-proteinases and a vast number of cytokines, in particular interleukins and the therapeutic target TNF-alpha. Overall, 389 gene-gene interactions between these genes could be retrieved through text mining. These interactions constitute a text mining network in which all but two genes are connected. The genes with the most connections are IL-6 (37), TNF-alpha (37) and IL-1 (33). We analyzed the regulatory regions of all the 42 genes and found overrepresented DNA-binding sites of 10 TFs (additional file 7). Amongst others, TFBS of NF-kappaB and AP-1 are significantly enriched, which is not surprising as both TF complexes play central roles in immune regulation and are proven to be involved in the pathogenesis of RA [10,46]. In the resulting GRN template these 10 TFs are linked to 31 genes by means of 67 TF-gene interactions. When constructing a linear model of the benchmarking GRN using our novel inference algorithm TILAR, 13 TF-gene interactions were discarded during the backward stepwise selection procedure, i.e. 54 TF-gene interactions remained in the model. LARS then provided model predictions for different degrees of network connectivity (in successive LARS steps representing the dependency on parameter *s*).

Next, we tested whether the inferred edges between genes exist or not in the text mining network containing 389 gene-gene interactions. For this purpose, we calculated the measures recall, precision and false positive rate (FPR) for different network connectivities. A plot of the precision versus the recall performance of a method (in case of LARS as a function of *s*) and the ROC (receiver operating characteristic) curve, where recall is plotted against FPR, are two widely used visualizations for performance evaluation [43,44]. The ROC analysis allows comparison of the inference quality against a random prediction by calculating the area under the curve (AUC), while an AUC(ROC) close to 0.5 corresponds to a random forecast.

**Figure 6:**
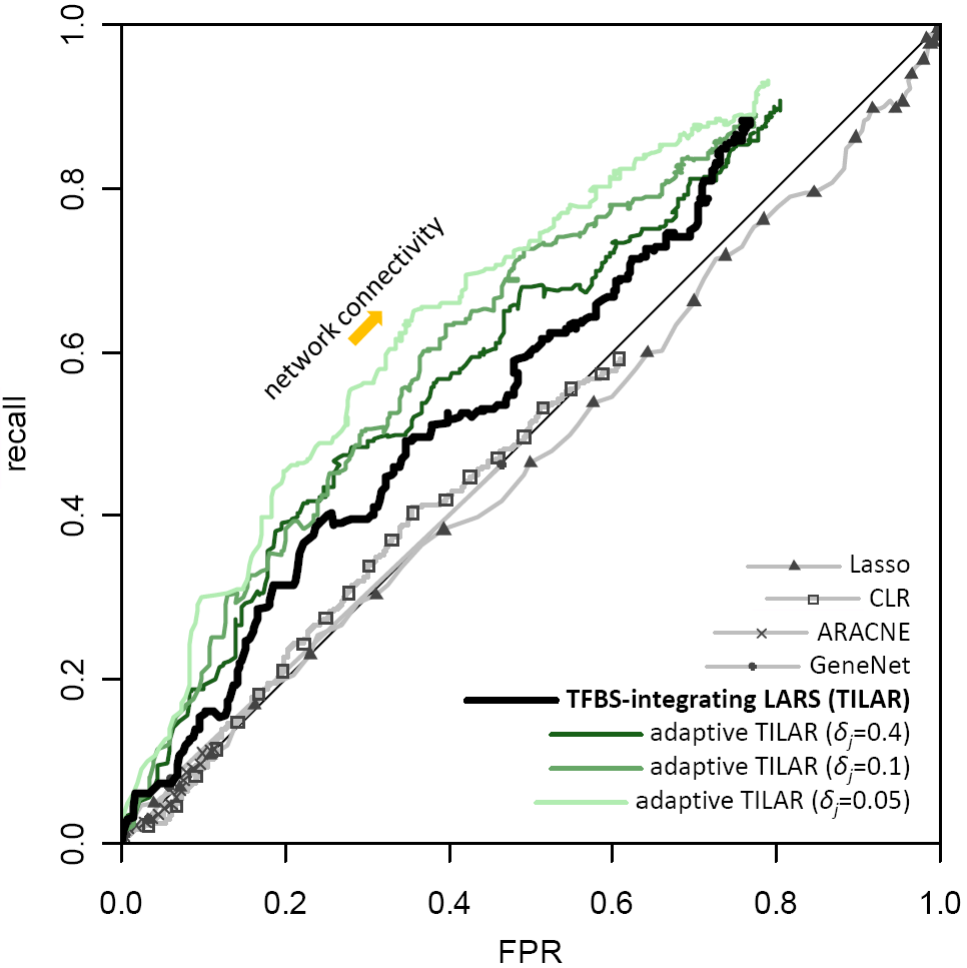
**ROC curves for the benchmarking gene regulatory network**. The better a method performs, the closer its curve will be to the upper-left corner. The black curve represents the rating of our method when including 54 of 67 predicted TF-gene interactions. Remarkably, TILAR not only outperforms CLR, ARACNE, GeneNet and the conventional Lasso, but can also be combined with the adaptive LARS if adequate prior knowledge on gene-TF interactions is available. Using both techniques in combination we could infer gene-gene relationships more reliably.

**Figure 7:**
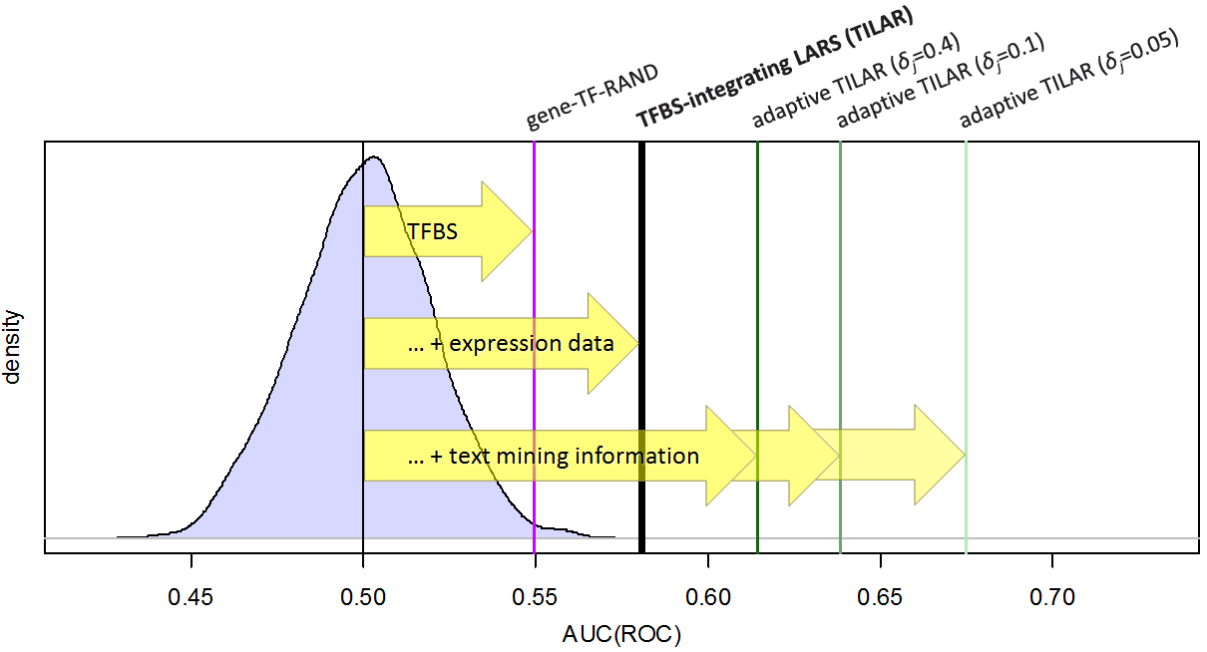
**Performance gain using integrative modeling**. The expected AUC(ROC) of a random prediction is normally distributed around 0.5 as calculated by 1,000 repeated runs of RAND. The gene-TF-RAND algorithm considers the GRN template information, but assigns gene-TF interactions randomly. In contrast, the TILAR algorithm utilizes gene expression data to infer gene-TF interactions, while only a subset of the predicted TF-gene interactions is included into the final model. This significantly increases the inference quality. However, the method could be further improved by considering text mining information on which genes possibly regulate TF activity (adaptive TILAR). The combined inference method allows to strike a balance between data-fit and confidence in such putative gene-TF interactions by means of the parameters in *δ*.

We utilized both recall-precision and ROC curves to assess and compare the performance of our algorithm and four different popular GRN inference methods: the conventional Lasso approach, CLR [47], ARACNE [48], and GeneNet [49] (see methods). While CLR and ARACNE use mutual information, GeneNet computes a partial correlation network. The resulting performance curves show that the proposed TILAR algorithm outperforms the other modeling algorithms (figure 6, additional file 8). When using AUC(ROC) as a single metric for benchmarking, the applied methods score as follows: Lasso – 0.478, ARACNE – 0.500, GeneNet – 0.503 and CLR – 0.504, whereas TILAR achieves an AUC(ROC) of 0.581. Next, we checked whether the algorithms performed significantly better than a random GRN prediction (RAND, see methods). We found, that the predictions of our approach were significantly better than RAND at the level *α *= 0.05 (*P-*value = 1.674e-05), while this was not the case for CLR, ARACNE, GeneNet and Lasso. Interestingly, gene-TF-RAND, another random algorithm that predicts gene regulatory interactions by including all 67 putative TF-gene interactions (i.e. the prior knowledge) into the model without considering the gene expression data (see methods), also yields a relatively high AUC(ROC) of 0.549 (*P*-value = 0.006). This suggests that TILAR performs well because of both the quality of TFBS predictions and data-fitting using LARS (figure 7).

Nevertheless, the AUC(ROC) of the TILAR method is still rather low. In our opinion this is not a general weakness of the modeling, but due to the fact that the information we used for model validation was obtained by text mining. This information is therefore incomplete and error-prone. A drawback is that the text mining network was constructed by searching through all biological literature and not only RA specific literature. Besides, text mining is obviously inappropriate to assess so far unknown regulatory interactions. In fact, the GRN model now provides new hypotheses that may be tested experimentally. However, there are several other factors that impede an accurate GRN reconstruction or an adequate performance evaluation. First, regulatory networks can exhibit large dynamic topological changes [50]. Thus, among the interactions in our network we might only identify the most robust ones or those that are most relevant in the specific study, implying that some other could be missed, even if they have been biologically demonstrated. Second, the contribution of different cell types is lost in the study. Third, the text mining network contains undirected gene-gene interactions. In contrast, the proposed modeling approach assigns directed interactions between genes and TFs, i.e. gene-gene interactions are only implicitly defined in the model. Fourth, the network model might be too simple to reliably infer more complex interactions. Here, we assumed that gene regulatory effects are linear and additive, and precluded auto-regulation, i.e. a gene is not allowed to control a TF for which it possesses a TFBS. The latter because the data were not well suited for inferring a dynamic model. Moreover, the inference algorithm is based on co-expression at the transcriptional level, even if the amount of mRNA may not correspond to the level and (regulatory) activity of the proteins. However, more data would be required to infer more accurate models.

### Adaptive TILAR – combined use of two techniques

Until now, we have shown that the proposed modeling approach, which utilizes gene expression data as well as TFBS predictions, performs fairly well in the reconstruction of GRNs. However, the method is not only an alternative to the adaptive LARS, but can also be used in combination with it (adaptive TILAR). The adaptive LARS [30] specified in equation (5) penalizes the coefficients *β *of equation (8) with weights *δ*, dependent on whether the coefficient receives prior knowledge. In this way, we are able to integrate prior knowledge on gene-TF interactions as well. The lower we set the weights *δ*_*j *_for the coefficients *β*_*j *_that represent putative gene-TF interactions, the more these interactions are *a priori *preferred to be in the model. The weights *δ *thus allow to trade off data-fit and confidence in prior knowledge. However, accurate knowledge on gene-TF edges is difficult to obtain due to their variable meanings. For instance, intermediary molecules may account for such relationships. Here, we again applied text mining to retrieve potential gene-TF links (see methods). This way, we found 71 gene-TF relationships for the benchmarking GRN (e.g. the well-known activation of NF-kappaB by IL-1). We then evaluated the adaptive TILAR algorithm with three different weights for the preferred coefficients. As a result, the inference quality increased considerably (figure 6 and 7). When setting *δ*_*j *_= 0.4 for the 71 preferred coefficients we obtained an AUC(ROC) of 0.615 (*P*-value = 1.768e-09), for *δ*_*j *_= 0.1 a value of 0.639 (*P*-value = 4.839e-13), and for a very low *δ*_*j *_= 0.05 a value of 0.675 (*P*-value<2.2e-16). An even lower *δ*_*j *_did not improve the result much. Thus, we can use information on TF-gene and gene-TF interactions to infer a GRN model, that predicts regulatory interactions between genes more reliably. Nevertheless, the true use of the combination of adaptive and TFBS-integrating LARS requires more investigation. For instance, the determination of the weights *δ *is not straightforward, as we might set different weights for each gene-TF interaction and even relatively high weights (i.e. *δ*_*j*_>1.0) if certain gene-TF relationships can be excluded *a priori*. However, this is beyond the scope of this paper.

## Conclusion

We developed TILAR – a method for deriving transcriptional regulatory networks from gene expression data by integrating TF binding predictions. The algorithm is also able to incorporate prior knowledge on the putative regulators of TF activity (adaptive TILAR). Our linear, additive modeling approach distinguishes genes and TFs in the network, and identifies the connections between them based on the fast LARS regression algorithm and specific constraints on the network structure. The major advantage of this modeling strategy is that only few model parameters are sufficient for a complex network, which is still easy to interpret. When applied on short-term gene expression profiles of RA patients treated with etanercept, the method uncovers molecular immunotherapeutic effects and thus provides testable hypotheses about the drugs' mechanisms of action. A closer look on the model revealed genes co-regulated by a common TF and TFs that regulate functionally related genes. Moreover, the reconstructed GRN exhibits a scale-free, self-regulating and massively parallel architecture.

We evaluated the inference quality using a text mining network and found that our modeling method outperforms all other algorithms tested. Notably, TILAR allows for a higher prediction accuracy than using just gene expression data or TF binding information alone. More efforts are needed to study different configurations of TILAR, e.g. we could analyze a larger DNA region for overrepresented TFBS, and to assess the benefit of combining this method with the adaptive LARS. Besides, further experiments need to be performed to verify specific interactions that were predicted by the model. However, even if significant theoretical and experimental challenges remain, we could demonstrate that organizing heterogeneous data and prior biological knowledge in systems biological models can strongly support the investigation of autoimmune diseases and their therapies. Supplementary materials including R codes are available at .

## Methods

### DNA microarray data pre-processing

We used the human DNA microarray dataset from Koczan *et al*. [13] including expression profiles of 19 etanercept-treated RA patients. Blood samples were taken for each patient before treatment as well as 72 and 144 hours after first application of etanercept. Transcriptional levels of PBMC were then measured using Affymetrix Human Genome U133A arrays. As for 2 patients the third time-point is missing, the dataset consists of 55 microarray experiments. In the applied Affymetrix microarrays most probesets include probes matching transcripts from more than one gene and probes which do not match any transcribed sequence. Therefore, we utilized a custom chip definition file (CDF), that is based on the information contained in the GeneAnnot database [15,51]. GeneAnnot-based CDFs are composed of probesets including only probes matching a single gene and thus allow for a more reliable determination of expression levels. We used version 1.4.0 of the custom CDF and the MAS5.0 algorithm to pre-process the raw probe intensities. Data normalization was performed by a loess fit to the whole data with *span *= 0.05 (using R package affy). Finally, the data processing yields mRNA abundances of 11,174 different genes.

### Filtering differentially expressed genes

The filtering aims to identify a subset of genes significantly up- or down-regulated within the first week of therapy. A widely used filter criterion is the (logarithmized) fold-change from baseline. However, a fixed fold-change threshold ignores the inherent structure of DNA microarray data. Therefore, we applied an MA-plot-based signal intensity-dependent fold-change criterion (MAID filtering) to select genes. The MAID filtering takes into account that the variability in the log fold-changes increases as the measured signal intensity decreases [52]. First, the filtering procedure calculates for each gene the values A and M, which are commonly used for visualizing microarray data in an MA-plot. A is the log signal intensity of a gene averaged over all patients, while M is the mean intensity log-ratio between the baseline levels and the expression levels at day 3 and 6, respectively. Then, the intensity-dependent variability in the data is estimated by computing the interquartile range (IQR) of the M values in a sliding window. Afterwards, an exponential function *f*(*x*) = *a*·e^-*bx*^*+c *is fitted to the IQR's by a non-linear robust regression, which in turn is used to calculate so-called MAID-scores by dividing each M value by *f*(A). As a consequence, the absolute value of a gene's MAID-score is higher, the more its expression level is altered after start of therapy. Furthermore, we assessed which genes are differentially expressed according to a paired *t-*test comparing the expression levels at day 3 and 6 versus baseline, respectively. Finally, we selected the genes having |MAID-score|>2.5 and *t*-test *P*-value < 0.05.

### GO analysis

Overrepresented GO terms were found using GOstats, a Bioconductor package written in R. Each GO term is tested whether it is significantly associated to the list of filtered genes out of the 11,174 measured genes. The analysis was performed for gene functional annotations of the biological process GO category.

### Identification of TF-gene interactions (GRN template)

TFBS were derived from the UCSC database build hg18 [53]. The database provides a TFBS conserved (tfbsConsSites) track, that contains the location and score of TFBS conserved in the human/mouse/rat alignment. The data are purely computational and were generated using position weight matrices (PWMs) for TFBS contained in the public Transfac Matrix and Factor databases created by Biobase. For the whole human genome 3,837,187 TFBS predictions associated to 258 different PWMs (184 unique TF identifiers) can be found in the tfbsConsSites track. We defined the regulatory region of each gene as the 1,000 bp up- and downstream of the TSS (as stated in GeneCards database 2.38). This specification is in agreement with current findings by the ENCODE pilot project which revealed that regulatory sequences are symmetrically distributed around the TSS with no bias towards upstream regions [54]. Then, we scanned the regulatory regions of the selected genes for overrepresented TFBS. In doing so, each TF is tested whether its binding site occurs in this region for more genes than would be expected by chance. To take into account the inherent redundancy of the Transfac database, a TF is supposed to regulate a gene (TF-gene interaction) if any PWM for this TF matches the DNA sequence at the gene's regulatory region. Using a hypergeometric test analyzing the TF binding predictions for all the 11,174 genes measured, we can identify a subset of TFs associated to the genes in the network at the significance level *α *= 0.05. This leads to a list of predicted TF-gene interactions that can serve as a template for GRN modeling.

### TFBS-integrating GRN inference (TILAR algorithm)

First, the expression levels of each gene were standardized so that they have variance 1 and mean 0. Given these data, we then defined a regression equation according to equation (8), while considering the full set of putative TF-gene interactions. Afterwards, we calculated all LARS estimates (steps) for this equation using the R package lars with default settings. Each LARS estimate specifies a subset of covariates, i.e. states which gene-TF interactions are present in the model and which are not (in the latter case the corresponding model parameter is set to zero). To select a single estimate, we chose the model that minimizes Mallows' Cp statistic [55], thereby preventing overfitting and ensuring sparseness. The whole procedure was then repeated in a backward stepwise selection scheme in which TF-gene interactions were iteratively eliminated (or reinserted) if this allowed for a model that exhibits a smaller residual sum of squares (RSS). In this way, a subset of TF-gene interactions was found. For the regression equation including this subset all possible Lasso estimates (see equation (4)) are provided by LARS. We then calculated for each LARS step the OLS fit using only the respective covariates (LARS/OLS hybrid [27]). Hence, we used LARS for variable selection, but not to estimate the model coefficients. Moreover, we evaluated the 10-fold cross-validation error (CV_error_) for each LARS/OLS solution and finally selected the most parsimonious model within 1 standard deviation from the CV_error _minimum (additional file 4). It should be noted that we used the Cp statistic as a crude selection criterion during the backward stepwise selection procedure, because the Cp is much faster to compute than the CV_error_.

To integrate prior knowledge on gene-TF interactions (as we did for the benchmarking GRN) we strictly followed the above learning strategy, except that we employed the adaptive variant of the LARS algorithm according to Zou [30]. The adaptive LARS assigns weights to each coefficient as written in equation (5). We penalized coefficients *β*_*j *_for which we have no prior knowledge with a neutral weight *δ*_*j *_= 1.0. If literature mining suggested a gene-TF interaction we penalized the corresponding coefficient with a smaller *δ*_*j *_(0.4, 0.1 and 0.05, respectively) to improve variable selection.

The learning strategy of the (adaptive) TILAR is summarized as follows:

1. Define *D *as the given (standardized) gene expression data

2. Define *P *as the given set of putative TF-gene interactions (GRN template)

3. Use *D *to specify regression equation *L *subject to *P *according to equation (8)

4. Solve *L *using (adaptive) LARS and calculate RSS(Cp_min_), i.e. the RSS of the LARS estimate that minimizes Cp

5. Optional: Perform a backward stepwise selection on *P*, i.e. iteratively and exhaustively remove or reinsert elements in *P *and repeat 3. and 4., and stop when a local minimum for RSS(Cp_min_) is found

6. Recompute the regression coefficients to *L *in terms of a LARS/OLS hybrid and return the most parsimonious estimate within 1 standard deviation of the 10-fold CV_error _minimum

### Performance evaluation

We used gene-gene interaction information for benchmarking. The software PathwayArchitect 2.0.1 was employed to automatically extract such gene-gene links from the literature. We retrieved only gene-gene interactions of context type "expression" and "regulation" as labeled by PathwayArchitect. To obtain putative gene-TF links (which were used for the adaptive TILAR) we also considered interactions of type "protein modification". The gene-gene information was applied to assess the inference quality of our and a total of four other easy-to-apply GRN inference methods, namely CLR, ARACNE, GeneNet, and the conventional Lasso, while we did not take into account the directions of the relationships. CLR, ARACNE and GeneNet are thought to build (undirected) gene association networks and have been implemented by use of the R packages minet and GeneNet. To compute the entire set of Lasso solutions to equation (3) we used the LARS modification (R package lars). All methods were run on standardized gene expression levels with default settings. Moreover, a random inference algorithm called RAND was implemented, which randomly assigns connections between genes until a fully connected network is formed. The RAND method was further adapted to infer networks of TF-gene and gene-TF interactions similar to the proposed modeling scheme (gene-TF-RAND). More specifically, gene-TF-RAND utilizes all the TF-gene interactions predicted by the TFBS overrepresentation analysis and randomly adds gene-TF edges to the network. As for TILAR, gene-TF interactions were not allowed when gene and TF were already connected by a TF-gene interaction, and gene-gene links result implicitly. The AUC(ROC) value of gene-TF-RAND was obtained by the mean of 1,000 repeated runs. Apart from that, we tested whether any inference technique performed significantly better than a random prediction. For this purpose, we calculated *P*-values which specify the probability that an AUC(ROC) value computed by RAND will be higher than the AUC(ROC) value of the particular inference algorithm. The *P*-values are calculated by 1 minus the cumulative probabilities, which are evaluated at the AUC(ROC) value of the respective method, of the normal distribution having the mean and standard deviation of 1,000 RAND-calculated AUC(ROC) values.

## Abbreviations

TF: transcription factor; GRN: gene regulatory network; RA: rheumatoid arthritis; TFBS: TF binding site; LARS: least angle regression; OLS: ordinary least squares; GO: gene ontology; TILAR: TFBS-integrating LARS; PBMC: peripheral blood mononuclear cells; MAID: MA-plot-based signal intensity-dependent fold-change criterion; TSS: transcription start site; Lasso: least absolute shrinkage and selection operator; FPR: false positive rate; ROC: receiver operating characteristic; AUC: area under the curve; CDF: chip definition file; IQR: interquartile range; PWM: position weight matrix; RSS: residual sum of squares.

## Conflict of interests

The authors declare that they have no competing interests.

## Authors' contributions

RGu and H-JT directed the study. MH carried out the analyses on the data and wrote the paper. RGu, RGo and RE assisted in interpretation of the results and contributed to writing the paper. All authors read and approved the final manuscript.

## Supplementary Material

Additional file 1 List of 83 genes with significant expression changes during first week of therapy. The table provides diverse types of information for each gene, e.g. Entrez ID, official full name and the calculated MAID-scores. Click here for file

Additional file 2 Filtering of genes regulated in response to etanercept therapy. (**A**) Superimposed MA-plot visualizing the applied gene filtering method. Here, gene expression levels measured 3 days after therapy onset are compared with baseline levels. The MAID filtering takes into account that the variability in the mean log-fold changes (M) depends on the mean log signal intensity (A). 37 genes showed an up- or down-regulation at day 3 (green). (**B**) In a similar manner, 57 genes were found higher or lower expressed at day 6 in comparison to baseline. In this way, 83 different genes were selected in total. (**C**) Mean time-courses of these 83 genes. 25 genes were found up- or down-regulated at day 3 (left), 45 at day 6 (middle) and 13 at day 3 and 6 (right). Click here for file

Additional file 3 Overrepresented terms of the GO biological process ontology. *P*-values were computed for each GO term based on the hypergeometric distribution. Only functional categories with *P*-value < 0.01 and where at least 3 out of 83 genes are associated ("Count") are shown. Click here for file

Additional file 4 Model selection using cross-validation. Training error (scaled by 10) and 10-fold CV_error _(RSS mean of 10 subsets) are shown for the LARS/OLS solutions of the first 300 LARS steps. The blue area represents the standard deviation of CV_error_. The red line shows the LARS step selected for the final model, i.e. the most parsimonious model within 1 standard deviation from the CV_error _curve minimum, for which 22 model parameters are non-zero. Click here for file

Additional file 5 Zip-archived Cytoscape session file of the reconstructed GRN. The network model contains predicted regulatory interactions of genes responsive to etanercept therapy in RA. A simplified visualization of the network is shown in figure 3, while detail views are shown in figure 5. Click here for file

Additional file 6 List of the 50 most frequently mentioned genes in the context of RA. 42 of these genes were measured in the dataset and used to evaluate the performance of our modeling approach. Click here for file

Additional file 7 Overrepresented binding sites for the benchmarking gene regulatory network. 10 transcription factors represented by 20 Transfac binding profiles were found to be enriched, providing 67 predicted TF-gene interactions in total. Click here for file

Additional file 8 Recall-precision curves for the benchmarking GRN. We evaluated the performance of different modeling strategies based on gene-gene relationships found by text mining. The black curve represents the rating of our method when including 54 of 67 predicted TF-gene interactions. The TILAR approach outperforms CLR, ARACNE, GeneNet and the conventional Lasso. When used in combination with the adaptive LARS we could further increase the inference quality. Click here for file
