# Supplementary figures and images for: Integrative modeling of transcriptional regulation in response to antirheumatic therapy
Source: BMC Bioinformatics. 2009 Aug 24;10:262. doi: 10.1186/1471-2105-10-262 (PMC2757030; doi:10.1186/1471-2105-10-262)

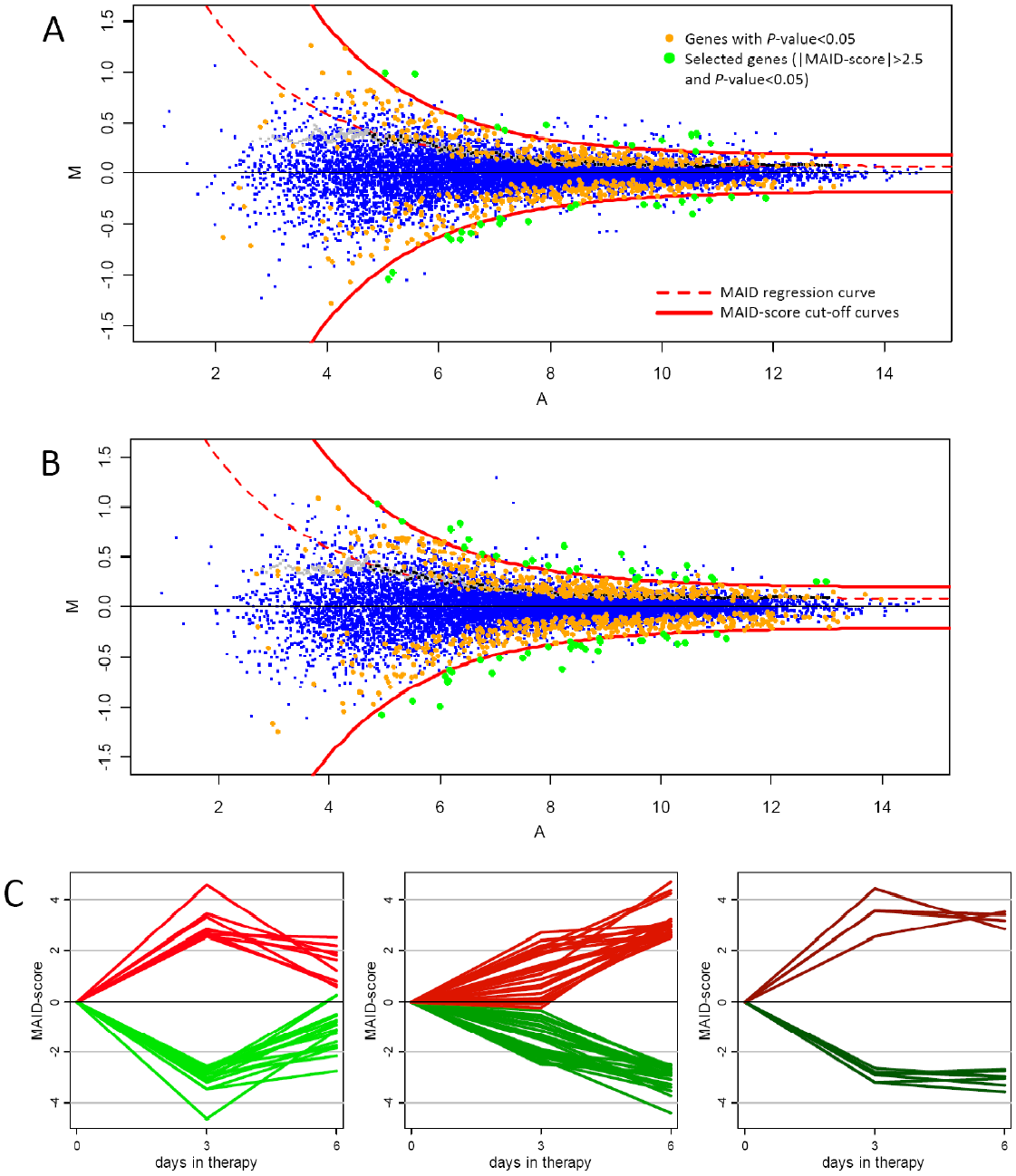

Supplement: Additional file 2 — Filtering of genes regulated in response to etanercept therapy. (A) Superimposed MA-plot visualizing the applied gene filtering method. Here, gene expression levels measured 3 days after therapy onset are compared with baseline levels. The MAID filtering takes into account that the variability in the mean log-fold changes (M) depends on the mean log signal intensity (A). 37 genes showed an up- or down-regulation at day 3 (green). (B) In a similar manner, 57 genes were found higher or lower expressed at day 6 in comparison to baseline. In this way, 83 different genes were selected in total. (C) Mean time-courses of these 83 genes. 25 genes were found up- or down-regulated at day 3 (left), 45 at day 6 (middle) and 13 at day 3 and 6 (right). [file 1471-2105-10-262-S2.png]

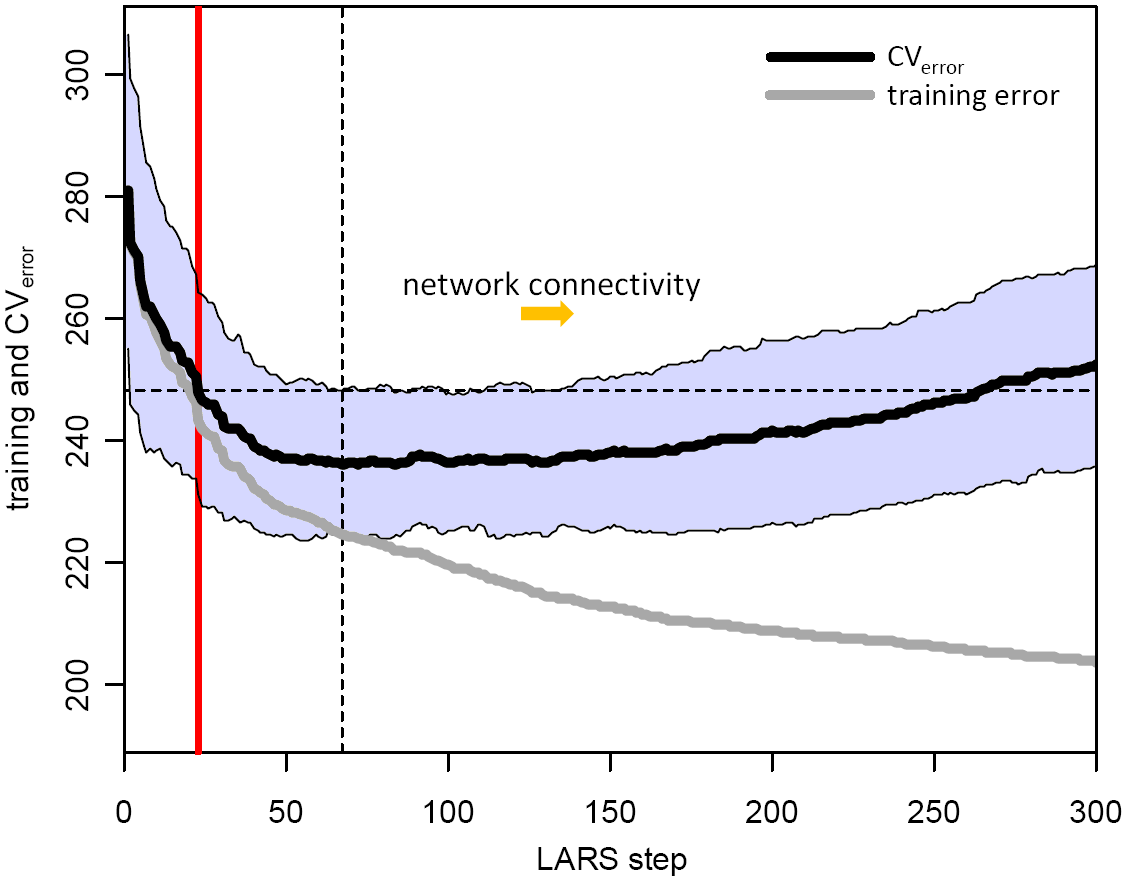

Supplement: Additional file 4 — Model selection using cross-validation. Training error (scaled by 10) and 10-fold CVerror (RSS mean of 10 subsets) are shown for the LARS/OLS solutions of the first 300 LARS steps. The blue area represents the standard deviation of CVerror. The red line shows the LARS step selected for the final model, i.e. the most parsimonious model within 1 standard deviation from the CVerror curve minimum, for which 22 model parameters are non-zero. [file 1471-2105-10-262-S4.png]

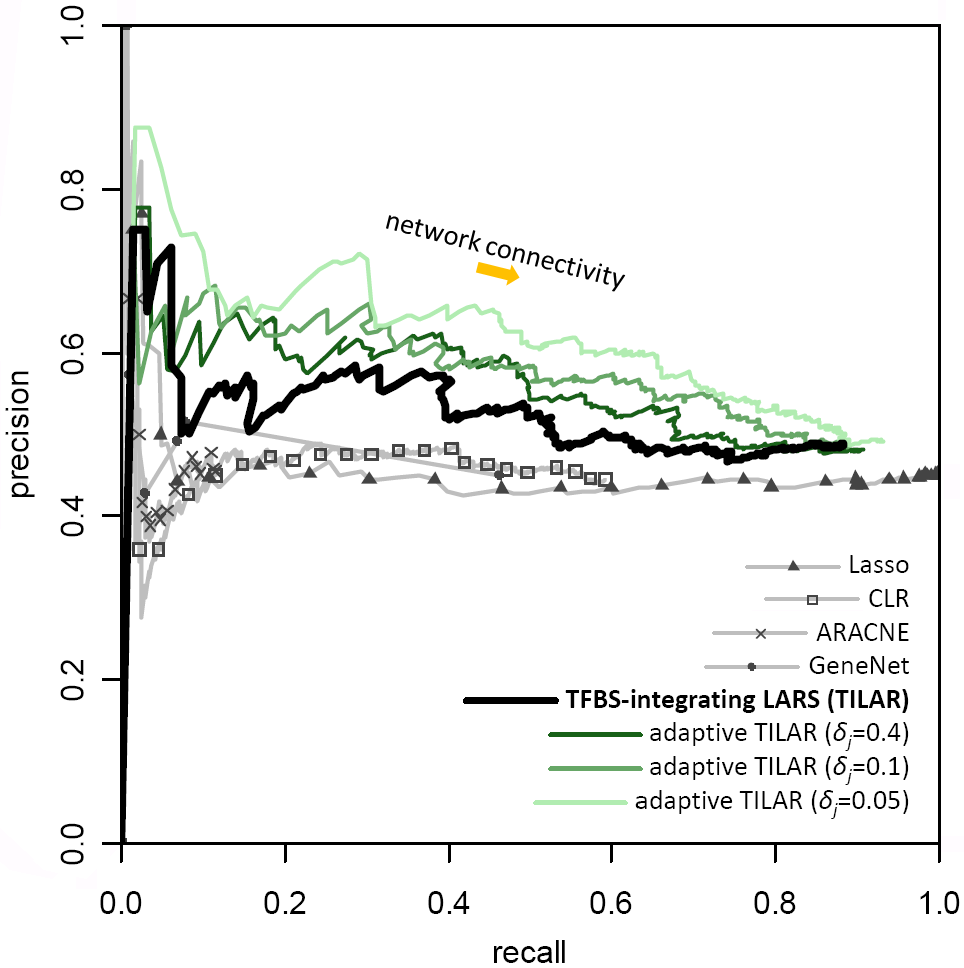

Supplement: Additional file 8 — Recall-precision curves for the benchmarking GRN. We evaluated the performance of different modeling strategies based on gene-gene relationships found by text mining. The black curve represents the rating of our method when including 54 of 67 predicted TF-gene interactions. The TILAR approach outperforms CLR, ARACNE, GeneNet and the conventional Lasso. When used in combination with the adaptive LARS we could further increase the inference quality. [file 1471-2105-10-262-S8.png]
